# The complete mitochondrial genomes of two sibling species of camellia weevils (Coleoptera: Curculionidae) and patterns of Curculionini speciation

**DOI:** 10.1038/s41598-019-39895-8

**Published:** 2019-03-04

**Authors:** Shou-ke Zhang, Jin-ping Shu, Yang-dong Wang, Ya-ning Liu, Han Peng, Wei Zhang, Hao-jie Wang

**Affiliations:** 1 State Key Laboratory of Tree Genetics and Breeding, Chinese Academy of Forestry, No. 1, Dongxiaofu Xiangshan Road, Haidian District, Beijing, 100091 P. R. China; 2 Research Institute of Subtropical Forestry, Chinese Academy of Forestry, No. 73, Daqiao Road, Fuyang District, Hangzhou, Zhejiang 311400 P. R. China

## Abstract

Complete mitochondrial genomes contain large and diverse datasets for species delineation. To better understand the divergence of the two morphologically indistinguishable weevil species in Curculionini, we first sequenced and compared their complete mitochondrial genomes. The complete mitochondrial genomes of *Curculio chinensis* and *Curculio* sp. were 19,713 bp with an A + T content of 76.61% and 19,216 bp with an A + T content of 76.85%, respectively. All 37 of the typical mitochondrial genes were determined in both species. The 13 protein sequences of the two species shared high homology (about 90%) except for ATP8 (73.08%). The differences in secondary structure of ATP8 were the number of possible proteins and nucleic acid binding sites. There were 22 and 15 mismatched base-pairs in the tRNA secondary structures from *C*. *chinensis* and *Curculio* sp., respectively. Maximum Likelihood and Bayesian analyses indicated that *Curculio* sp. is a novel species closely related to *C*. *chinensis*. The divergence time estimation suggests that Cryptorhynchinae and Curculionini lines diverged in the Cenozoic Period, while *C*. *chinensis* and *Curculio* sp. diverged at 6.7079 (95% CI 5–13) Mya. This study demonstrates the utility of using complete mitochondrial gene sets for phylogenetic analysis and enhances our understanding of the genetic basis for the evolution of the Curculionini.

## Introduction

The camellia weevil is a host-specific predator of the seeds of *Camellia oleifera* Abel. and *Camellia sinensis* (L.) O. Ktze. (Theaceae) which have been widely cultivated as important economic trees in China, and it is often identified as *Curculio chinensis* Chevrolat^1–4^. The species exhibits low levels of regional diversity and low degrees of morphological differentiation. However, a few geographic populations are phylogenetically distinct, and show significant variation related to *Camellia* host isolation (*C*. *oleifera* and *C*. *sinensis*)^4^. Camellia weevils in species complexes are often difficult or impossible to identify using morphological characteristics of the larvae. It is impractical to identify camellia weevils by rearing larvae to adults because the larvae are long-lived and difficult to rear when removed from the seed^1,2^. Molecular identification has proven to be reliable and more effective^5^.

Mitochondria exist in plant and animal cells and are inherited maternally. They are associated with metabolism, life cycle, and apoptosis and are abundant in energy consuming tissues related to essential biological functions^6,7^. Genetic data from mitochondria are commonly used to study evolutionary relationships. Useful information for evolution studies continues to be found in mitochondrial gene markers^8–11^. Relatively high mutation rates and low recombination frequencies make mitochondrial genomes especially useful for evolutionary studies^12–15^. However, shorter mitochondrial gene sequences often do not provide adequate phylogenetic resolution^16–18^. For example, our previous phylogenetic analysis based on the cytochrome oxidase subunit I (COX1) gene revealed the presence of a novel *Curculio* sp. in China feeding on *Camellia* seed. *Curculio* sp. is closely related to *C*. *chinensis*. However, the information provided by COX1 alone did not provide sufficient support for naming the new species^4^. Fortunately, complete mitochondrial genomes have become available for the identification of animal species^19^. Also, mitochondrial genomes have been used for phylogenetic analyses in many species, and especially in recent DNA-barcoding studies^15,20,21^.

High-throughput sequencing methodologies such as whole genome sequencing (WGS) and next generation sequencing (NGS) provide complete mitochondrial genomes for phylogenetic analyses and the replacement of earlier markers such as COX1, COX2, and NAD genes. However, the ability to resolve closely-related lineages with mitochondrial genomes remains controversial^7,13,15,22,23^. Generally, a complete mitochondrial insect genome is a circular-DNA molecule comprising 15–21 kbp of DNA. It features a relatively conserved gene content including 13 protein-coding genes (PCGs), 2 ribosomal RNA (rRNA) genes, and 22 transfer RNA (tRNA) genes, in addition to an A + T-rich region^6,15,18,23,24^. Insect mitochondrial genome gene sequences, and particularly protein coding genes, are relatively conserved^8,9^. The 13 PCGs of insect mitochondria are useful phylogenetic markers^25,26^ and have been used for phylogenetic analyses of species in the Diptera, Coleoptera and Lepidoptera^7,15,22,24,27^. In addition, insect mitochondrial tRNAs exhibit rearrangement phenomena^28^, while other genes rarely exhibit such rearrangements^29^. The transposition genotypes of tRNAs, in addition to the combinations of six tRNAs between the mitochondrial ND3 and ND5 genes provide additional informative characters^23,30^. Mitochondrial genomes are therefore excellent molecular markers for studies of species delineation, population genetics and evolution.

The increasing availability of mitochondrial genomes, combined with next-generation sequencing technologies^31–33^, have enabled detailed comparative phylogenetic analyses of many species complexes^34,35^ and the major taxonomic groups of several insect orders have undergone diagnostic revisions^5,22,26,28,36^. In this study, we obtained the complete mitochondrial genomes from *C*. *chinensis* and a novel *Curculio* sp., and compared them with the mitochondrial genomes of other Curculionid species. These analyses provided insight into their genome evolution as well as the phylogeny of the Curculionidae.

## Results and Discussion

### Features of the sequenced mitochondrial genomes

Coleoptera mitochondrial genomes have relatively simple structures and lack spacers and introns. With this tight gene arrangement, genetic rearrangement, inversions, and translocations occur infrequently during the mutation process^6,37^. The complete mitochondrial genome sequences of *C*. *chinensis* and the *Curculio* sp. were 19,713 bp and 19,216 long, respectively (GenBank accessions MG728094 and MG728095) (Table S1). The genomes of both species contained all 37 typical animal mitochondrial genes, including 13 protein-coding genes, 22 tRNA genes, and two rRNA genes (Fig. S1). The A + T-rich region generated reliable sequence data in both species which was high (>75%) compared to that found in other mitochondrial genomes sequenced using NGS^15^. No gene rearrangement was observed in either species compared with the putative ancestral and sibling superfamily arrangement (Fig. S1). This is consistent with the lack of rearrangement found in all sequenced Curculionini species^8,11^.

The mitochondrial genome of *C*. *chinensis* has intergenic spacers with lengths ranging from 1 to 103 bp in 24 different locations. Seven pairs of genes overlap with each other, with overlap lengths ranging from 1 to 17 bp. Eight pairs of genes are directly adjacent to one another, which include the pairs rrnL-trnV and trnV-rrnS (Table 1). The mitochondrial genome of *Curculio* sp. has intergenic spacer lengths ranging from 1 to 148 bp in 26 different locations. Eight pairs of genes overlap with each other, with overlap lengths ranging from 1 to 7 bp. Nine pairs of genes were directly adjacent one another, which included the pairs rrnL-trnV and trnV-rrnS (Table 2). In both species, the longest intergenic spacer was located between trnS2 and NAD1. The longest overlapping regions were located between trnF and NAD5 (Tables 1 and 2). The intergenic and overlapping regions of these two species were similar to the mitochondrial genomes of most other insects. Similarly, no gene rearrangement was found in either species compared to genomes from Coleoptera species that have experienced frequent gene rearrangement^23,28^.

**Table 1:** Annotation of the *Curculio chinensis* mitochondrial genome.

Gene	Strand	Position	Length (bp)	Initiation codon	Stop codon	Anticodon	Intergenic nucleotide (bp)
trnI	N	1–65	65			GAT	3,018
trnQ	J	3,084–3,152	69			TTG	1
trnM	N	3,154–3,221	68			CAT	
NAD2	N	3,222–4,232	1,011	ATT	TAA		14
trnW	N	4,247–4,310	64			TCA	−1
trnC	J	4,310–4,375	66			GCA	2
trnY	J	4,378–4,441	64			GTA	−8
COX1	N	4,434–5,978	1,545	ATT	TAA		−5
trnL2	N	5,974–6,038	65			TAA	
COX2	N	6,039–6,722	684	ATT	TAA		1
trnK	N	6,724–6,794	71			CTT	2
trnD	N	6,797–6,861	65			GTC	
ATP8	N	6,862–7,020	159	ATT	TAA		−7
ATP6	N	7,014–7,688	675	ATG	TAA		4
COX3	N	7,693–8,480	788	ATG	TA (A)		−1
trnG	N	8,480–8,543	64			TCC	
NAD3	N	8,544–8,897	354	ATT	TAG		−2
trnA	N	8,896–8,962	67			TGC	
trnR	N	8,963–9,024	62			TCG	−2
trnN	N	9,023–9,086	64			GTT	
trnS1	N	9,087–9,153	67			TCT	7
trnE	N	9,161–9,224	64			TTC	
trnF	J	9,225–9,289	65			GAA	−17
NAD5	J	9,273–11,000	1,728	ATT	TAA		9
trnH	J	11,010–11,072	63			GTG	−3
NAD4	J	11,070–12,408	1,339	ATG	T (AA)		−7
NAD4l	J	12,402–12,695	294	ATG	TAA		2
trnT	N	12,698–12,762	65			TGT	
trnP	J	12,763–12,828	66			TGG	2
NAD6	N	12,831–13,334	504	ATT	TAA		
COB	N	13,335–14,474	1,140	ATG	TAA		
trnS2	N	14,475–14,541	67			TGA	103
NAD1	J	14,645–15,571	927	ATA	TAG		25
trnL1	J	15,597–15,661	65			TAG	9
rrnL	J	15,671–16,944	1,274				22
trnV	J	16,967–17,032	66			TAC	
rrnS	J	17,033–17,821	789				1,891

**Table 2:** Annotation of the *Curculio* sp. mitochondrial genome.

Feature	Strand	Position	Length (bp)	Initiation codon	Stop codon	Anticodon	Intergenic nucleotide (bp)
trnI	N	1–65	65			GAT	2,007
trnQ	J	2,073–2,141	69			TTG	2
trnM	N	2,144–2,211	68			CAT	
NAD2	N	2,212–3,222	1,011	ATG	TAA		15
trnW	N	3,238–3,301	64			TCA	−1
trnC	J	3,301–3,366	66			GCA	2
trnY	J	3,369–3,432	64			GTA	−8
COX1	N	3,425–4,969	1,545	ATT	TAA		−5
trnL2	N	4,965–5,029	65			TAA	21
COX2	N	5,051–5,713	663	ATG	TAA		1
trnK	N	5,715–5,785	71			CTT	2
trnD	N	5,788–5,851	64			GTC	
ATP8	N	5,852–6,010	159	ATT	TAA		−7
ATP6	N	6,004–6,678	675	ATG	TAA		3
COX3	N	6,682–7,469	788	ATG	TA (A)		−1
trnG	N	7,469–7,536	68			TCC	6
NAD3	N	7,543–7,890	348	ATT	TAG		−2
trnA	N	7,889–7,956	68			TGC	
trnR	N	7,957–8,019	63			TCG	−2
trnN	N	8,018–8,080	63			GTT	
trnS1	N	8,081–8,147	67			TCT	6
trnE	N	8,154–8,217	64			TTC	
trnF	J	8,218–8,282	65			GAA	−17
NAD5	J	8,266–9,993	1,728	ATT	TAA		9
trnH	J	10,003–10,065	63			GTG	−3
NAD4	J	10,063–11,401	1,339	ATG	T (AA)		−7
NAD4l	J	11,395–11,688	294	ATG	TAA		2
trnT	N	11,691–11,755	65			TGT	
trnP	J	11,756–11,820	65			TGG	2
NAD6	N	11,823–12,326	504	ATT	TAA		
COB	N	12,327–13,466	1,140	ATG	TAA		
trnS2	N	13,467–13,533	67			TGA	148
NAD1	J	13,682–14,608	927	ATA	TAG		25
trnL1	J	14,634–14,698	65			TAG	5
rrnL	J	14,704–15,977	1,274				29
trnV	J	16,007–16,071	65			TAC	
rrnS	J	16,072–16,856	785				2,359

### Base composition

AT-skew, GC-skew, A + T content, and AT and GC asymmetries, are often used to assess the nucleotide-compositional differences of mitochondrial genomes^38^. The mitochondrial genomes of *C*. *chinensis* and the *Curculio* sp. were biased in nucleotide composition ((A + T)% > (G + C)%) across the whole genome, although the numbers of PCGs (n = 13) and rRNA genes (n = 22) were consistent with the genomes from other insects^5,24^. The A + T content of the whole genome was 76.61% for *C*. *chinensis* (40.16% A, 36.45% T, 9.89% G and 13.50% C) (Tables 3), and 77.08% for *Curculio* sp. (40.84% A, 36.24% T, 9.14% G and 13.79% C) (Table 4). The A + T content of all PCGs in *C*. *chinensis* ranged from 69.58% (COX1) to 83.38% (NAD6) (Table 3), and in *Curculio* sp. ranged from 69.84% (COX1) to 84.33% (NAD6) (Table 4). Most of the AT-skews of the two *Curculio* species were negative except COX2 and ATP8. Most of the GC-skews of both species were negative as well, indicating that the PCGs contained a higher percentage of T and C than A and G, as reported for most other insects^22,38^.

**Table 3:** Base composition of the mitochondrial genome of *Curculio chinensis*.

Region	A%	C%	G%	T%	A + T%	G + C%	AT skew	GC skew
Whole genome	40.16	13.5	9.89	36.45	76.61	23.39	0.048	−0.154
NAD2	35.41	14.54	8.61	41.44	76.85	23.15	−0.079	−0.256
COX1	32.62	16.83	13.59	36.96	69.58	30.42	−0.062	−0.106
COX2	37.28	16.52	10.23	35.96	73.25	26.75	0.018	−0.235
ATP8	42.14	13.21	4.4	40.25	82.39	17.61	0.023	−0.5
ATP6	36.3	16.59	8.89	38.22	74.52	25.48	−0.026	−0.302
COX3	36.42	16.62	11.42	35.53	71.95	28.05	0.012	−0.186
NAD3	36.72	12.43	8.19	42.66	79.38	20.62	−0.075	−0.205
NAD5	29.75	8.1	13.08	49.07	78.82	21.18	−0.245	0.235
NAD4	28.75	8.14	12.47	50.63	79.39	20.61	−0.276	0.21
NAD4l	29.25	5.1	13.95	51.7	80.95	19.05	−0.277	0.464
NAD6	39.09	8.53	6.75	45.63	84.72	15.28	−0.077	−0.117
COB	35.44	15.61	10.44	38.51	73.95	26.05	−0.042	−0.199
NAD1	27.4	8.63	15.1	48.87	76.27	23.73	−0.281	0.273
rrnL	36.81	6.36	13.34	43.49	80.3	19.7	−0.083	0.355
rrnS	37.01	8.11	15.84	39.04	76.05	23.95	−0.027	0.323

**Table 4:** Base composition of the mitochondrial genome of *Curculio* sp.

Region	A%	C%	G%	T%	A + T%	G + C%	AT skew	GC skew
Whole genome	40.84	13.79	9.14	36.24	77.08	22.92	0.06	−0.203
NAD2	36.99	13.85	7.72	41.44	78.44	21.56	−0.057	−0.284
COX1	32.04	16.31	13.85	37.8	69.84	30.16	−0.082	−0.082
COX2	37.86	17.5	11.01	33.63	71.49	28.51	0.059	−0.228
ATP8	44.03	12.58	3.14	40.25	84.28	15.72	0.045	−0.6
ATP6	36.15	15.11	8.89	39.85	76	24	−0.049	−0.259
COX3	36.17	15.61	11.55	36.68	72.84	27.16	−0.007	−0.15
NAD3	34.48	15.23	8.05	42.24	76.72	23.28	−0.101	−0.309
NAD5	29.11	8.97	13.43	48.5	77.6	22.4	−0.25	0.199
NAD4	29.05	9.19	12.32	49.44	78.49	21.51	−0.26	0.146
NAD4l	29.25	5.78	13.27	51.7	80.95	19.05	−0.277	0.393
NAD6	39.88	8.53	7.14	44.44	84.33	15.67	−0.054	−0.089
COB	34.21	16.05	10.7	39.04	73.25	26.75	−0.066	−0.2
NAD1	26.11	9.06	16.18	48.65	74.76	25.24	−0.302	0.282
rrnL	35.79	6.36	13.81	44.03	79.83	20.17	−0.103	0.37
rrnS	35.92	8.15	15.8	40.13	76.05	23.95	−0.055	0.319

### Protein-coding genes, codon usage, and protein conformance rates

**Figure 1:**
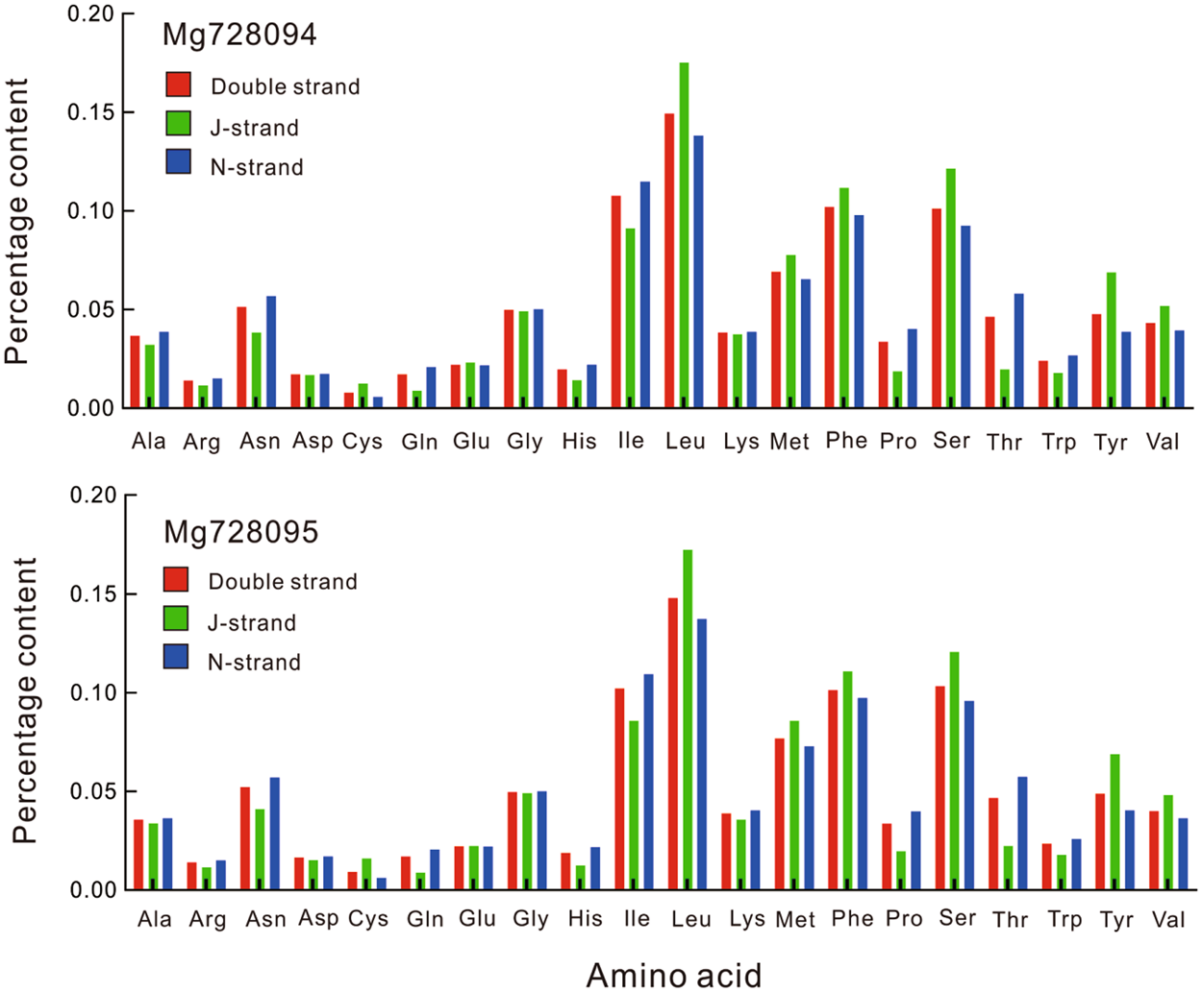
Percentage of each amino acid for proteins coded by PCGs in the two newly mitochondrial genomes of *Curculio chinensis* (MG728094) and *Curculio* sp. (MG728095).

In the mitochondrial genomes of both *C*. *chinensis* and *Curculio* sp., nine of the 13 protein-coding genes were located on the majority strand (N-strand), while the other four protein-coding genes were located on the minority strand (J-strand) (Tables 1 and 2). In the *C*. *chinensis* mitochondrial genome, the total length of protein-coding genes was 11,148 bp, accounting for 56.55% of the whole genome. The total length of the protein-coding genes of *Curculio* sp. was 11,121 bp, accounting for 57.87% of the whole genome (Tables 1 and 2). PCGs contained Leu residues in the highest abundance, followed by Ile, Phe and Met. The four amino acids had the highest use frequency (Fig. 1), similar to other insect mitochondrial genomes^36,39^.

In the mitochondrial genomes of both species, all PCGs start with the conventional initiation codons (ATN) as seen in other insects^40^. In *C*. *chinensis* PCGs, only one gene (NAD1) used ATA, seven used ATT, and five used ATG. In contrast, in the PCGs of *Curculio* sp., only one gene (NAD1) used ATA, while five and seven PCGs started with ATT and ATG, respectively (Tables 1 and 2). In both the *C*. *chinensis* and *Curculio* sp. mitochondrial genome, nine PCGS used TAA as the stop codon, and the NAD1 and NAD3 genes used TAG, while the COX3 and NAD4 genes used an incomplete stop codon T (Tables 1 and 2). The usage of incomplete stop codons in PCGs is common in invertebrate mitochondrial genomes^5,41^.

**Figure 2:**
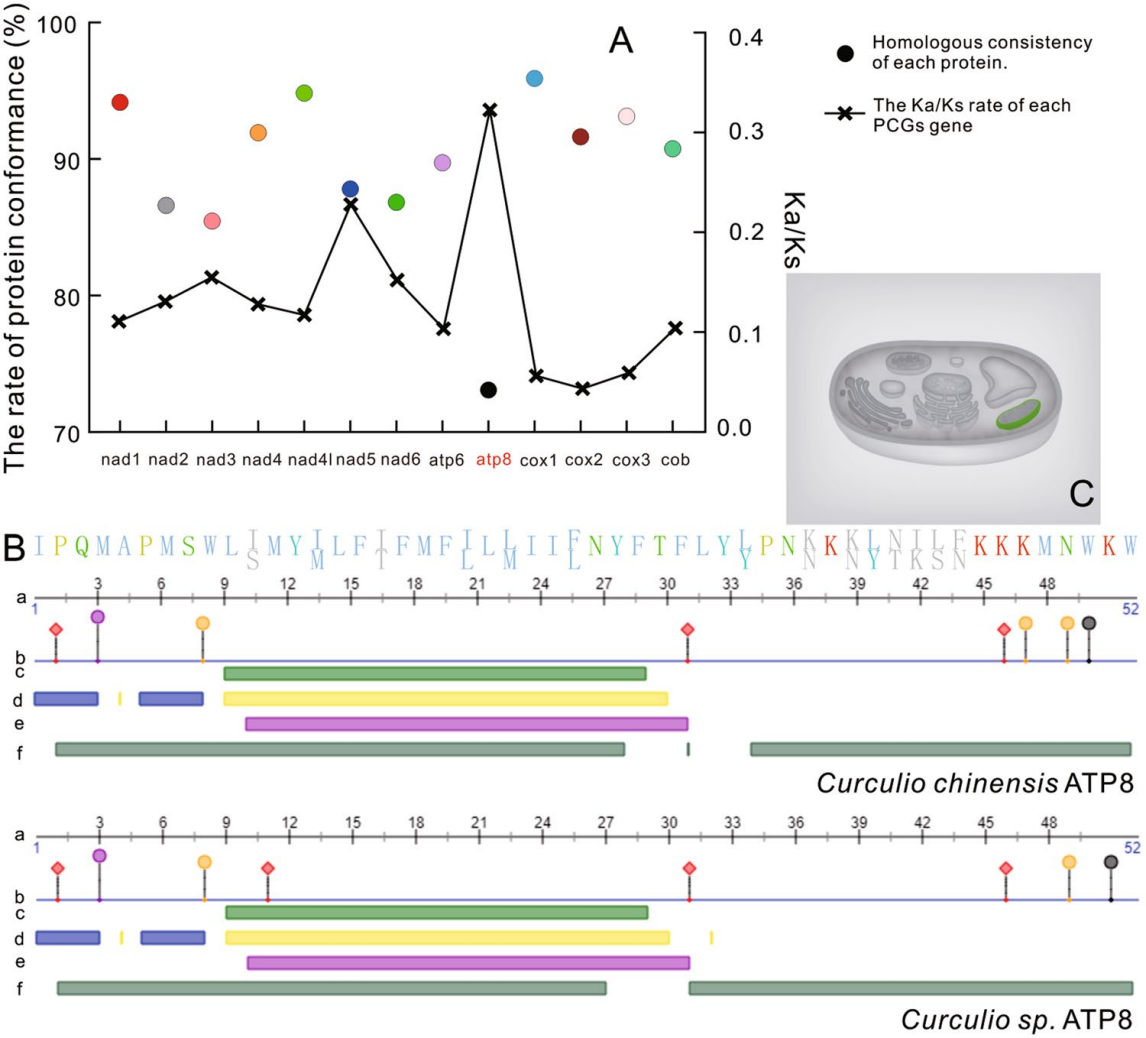
Protein conformance of each protein coding gene (PCG) in the mitochondrial genomes of *Curculio chinensis* and *Curculio* sp. The K_a_/K_s_ values of each PCG represented the ratios of non-synonymous substitutions (K_a_) to synonymous substitutions (K_s_) (**A**). ATP8 protein structure prediction (**B**). a: Protein sequence; b: Protein-protein binding; c: Secondary structure; d: Solvent accessibility; e: Transmembrane helix; f: Disordered region. (**C**) The predicted cellular compartment, mitochondrial membrane is highlighted in green in a schematic of a eukaryotic cell.

We calculated the homologous consistency of the 13 protein sequences of the two species as one group. Except for the ATP8 sequences that exhibited a value of 73.08%, the rest of the sequences had values of about 90% (Fig. 2A). Ratios of K_a_/K_s_ values for each PCG in the two species showed that ATP8 had the largest ratio (0.3194) among all proteins (Fig. 2A). Two genes, ATP6 and ATP8 are the core subunits of Complex V, which consists of F_0_ and F_1_, and the two genes are directly involved in ATP synthesis^42–44^.

We further characterized the ATP8 proteins from both genomes and predicted their structures (Fig. 2B), since this was the most variable gene of the 13 PCGs. The ATP8 protein sequences in both species contained 52 amino acids. The *C*. *chinensis* ATP8 protein structure contained three possible protein binding sites and five possible nucleic acid binding sites (Fig. 2B). Across sites 9–29, there was a region that might produce a spiral structure (Fig. 2B). In the entire chain of the *C*. *chinensis* ATP8 protein, there were three disordered areas, two exposed regions, two buried regions, and one transmembrane helix region (Fig. 2B). The *Curculio* sp. ATP8 protein structure has four possible protein binding sites and four possible nucleic acid binding sites (Fig. 2B). Like the *C*. *chinensis* ATP8 protein structure, there was also a region that might produce a spiral structure across the sites comprising 9–29 (Fig. 2B). In the whole chain of the *Curculio* sp. ATP8 protein, there were two disordered areas, two exposed regions, three buried regions, and one transmembrane helix region (Fig. 2B). The SOPMA analysis of the ATP8 secondary structure revealed clear structural differentiation between the two species. The alpha helix represented 38.46% and 59.62% of the structures of *C*. *chinensis* and *Curculio* sp., respectively, while the extended strand regions were 13.08% and 11.54%, the beta turn regions were 9.62% and 1.92%, and the random coil accounted for 28.85% and 26.92%, respectively. Adaptive evolution of ATP synthase can occur among species living in different ecological niches^39,44,45^. Thus, we speculated that modifications in the sequence and conformation of ATP8 structures could affect the assembly and function of Complex V, and consequently modulate its ability to produce ATP in *Curculio* weevils.

### Phylogenetic relationships and comparison of divergence times

**Figure 3:**
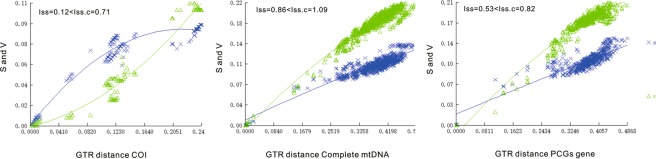
Saturation plots for (from left to right) COX1, the complete mtDNA genomes and PCGs. The plot showed uncorrected pairwise divergences in transitions (s) and transversions (v) against divergences calculated using the GTR model. Blue: transitions; Green: transversions.

In previous studies, mitochondrial sequence length variation was low, resulting in minimal alignment ambiguity that was not investigated further^23^. We calculated saturation plots for COX1, complete mtDNA genomes, and the PCGs before we used these to build a phylogenetic tree. The plots showed uncorrected pairwise divergences in transitions (s) and transversions (v) against divergences calculated with the GTR model, and none of the three genes had reached saturation (Fig. 3).

**Figure 4:**
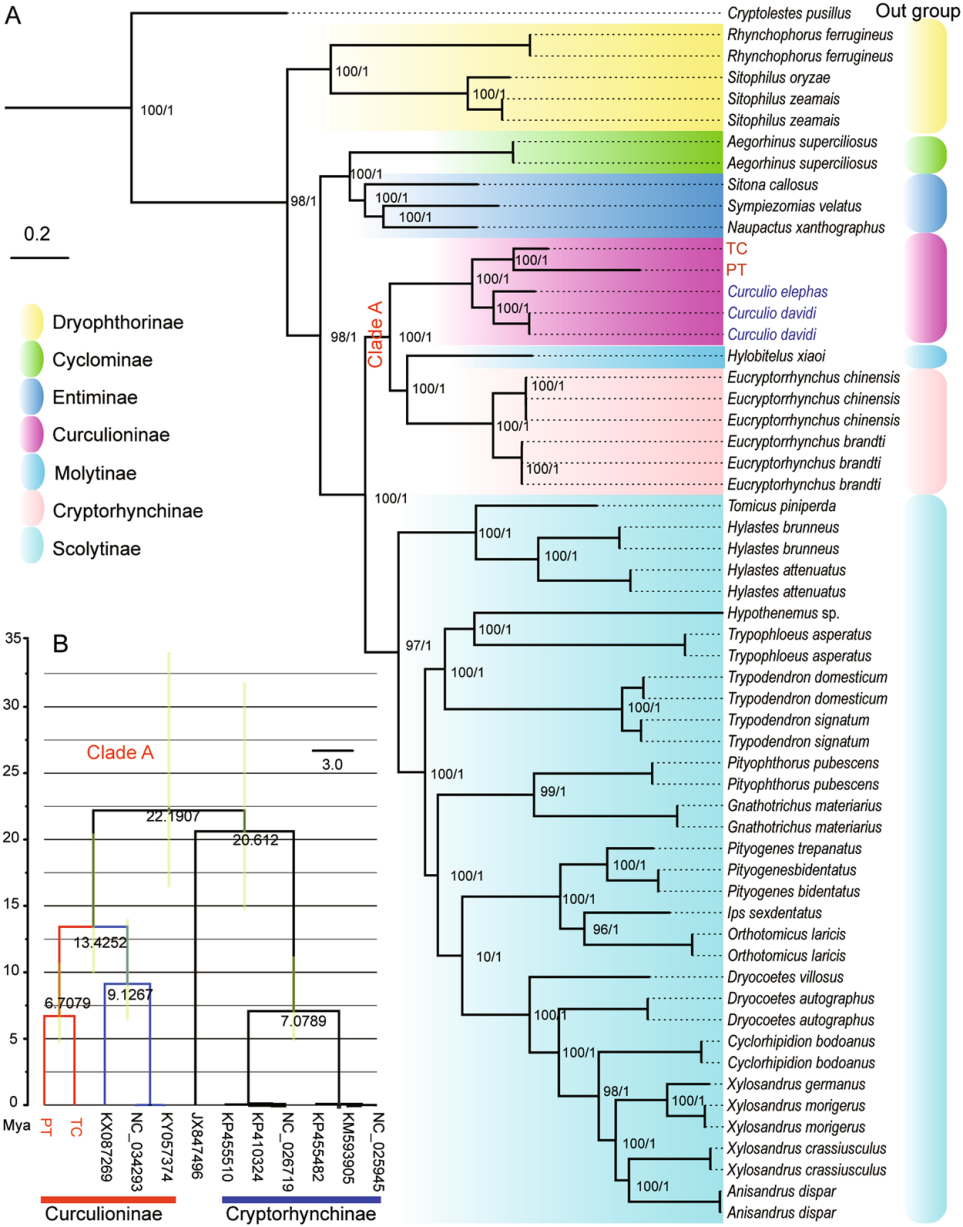
Maximum Likelihood and Bayesian phylogenetic tree based on protein sequences of 13 PCGs from the mitochondrial genomes of 57 species (**A**). Bootstrap supports of >90% for ML (upper) and posterior probabilities of >90% for BI (lower) were indicated around branches. PT: *Curculio chinensis* (MG728094) and TC: *Curculio* sp. (MG728095); The colors represent different subfamilies. Timescale for Clade A evolution and comparison of divergence times based on the 13 PCGs (**B**). The green horizontal bars represent 95% credibility intervals.

Maximum Likelihood and Bayesian phylogenetic trees were constructed based on the protein sequences of the 13 PCGs from the mitochondrial genomes of 57 Curculionid species (Fig. 4A). The results supported substantially our previous hypothesis that *Curculio* sp. is an undescribed species which was closely related to *C*. *chinensis*^4^. Our study showed that the Curculioninae fall into the diverse “CMC” clade (Curculioninae + Molytinae + Cryptorhynchinae), which is consistent with previous results^32,46–50^. Additionally, the genus *Curculio* is a typical member of Curculionini in Curculioninae, which showed close affinity with the subfamilies Molytinae and Cryptorhynchinae. Family-level studies have been used to estimate phylogenetic divergence times for Coleoptera using molecular data^12,25,48,50^. The studies suggest that the last common ancestor of Coleoptera occurred in the Permian period (253–297 Mya). The Cucujiformia species first occurred in the Triassic period (200–250 Mya). However, the Curculionidae might have first appeared in the Cretaceous period (60–150 Mya)^12,48,50^. Our data suggest that the Cryptorhynchinae + Molytinae and Curculioninae diverged at 22.1907 (95% credibility interval 16–35) Mya in the Cenozoic period (0–60 Mya), while *C*. *chinensis* and *Curculio* sp. diverged at 6.7079 (95% credibility interval 5–13) Mya (Fig. 4B). The divergence time between the two host plants, *C*. *oleifera* and *C*. *sinensis* was about 5–6 million years ago, which is consistent with the formation time of the earliest camellia fossils found in the tertiary stratum in Japan^51^. The geographic isolation of *Camellia* hosts might have played a role in the differentiation of camellia weevils.

### Transfer RNA and ribosomal RNA genes

**Figure 5:**
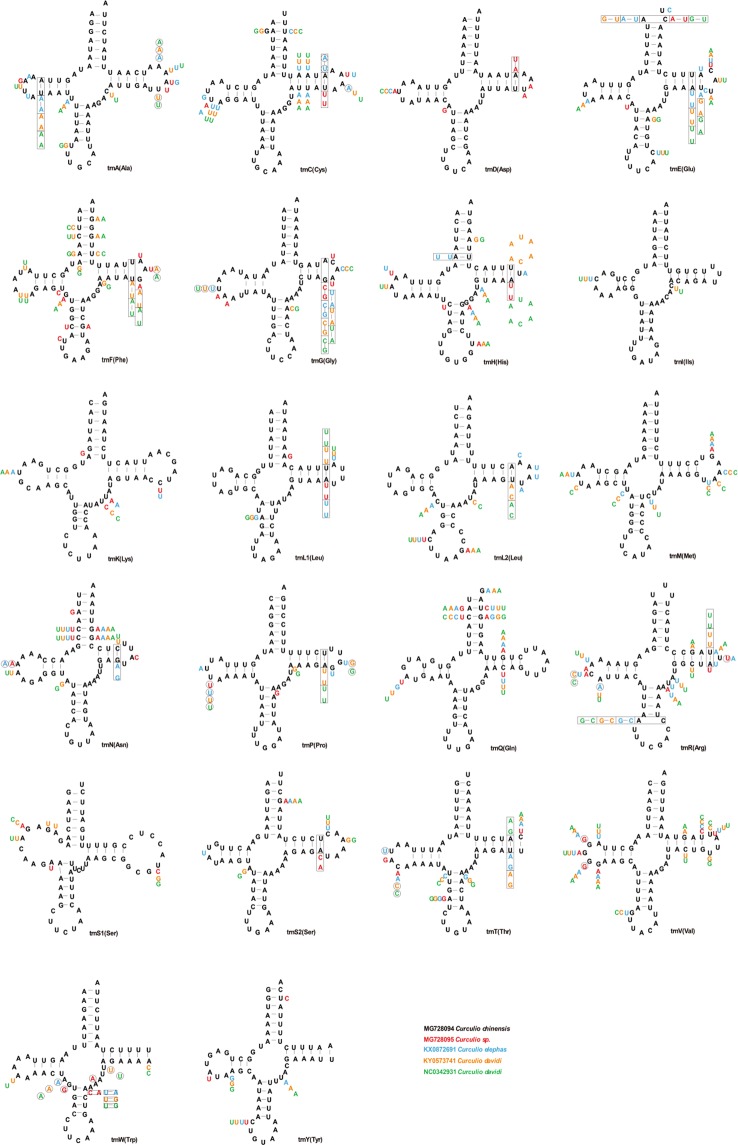
Comparison on the secondary structure of tRNA genes in Curculionini mitochondrial genomes. The secondary structures were drawn from tRNA genes of *Curculio chinensis*. Variations at each site in other four species of Curculionini were indicated near the corresponding nucleotide. Each species was marked by a unique color as shown in the legend at the bottom right of the figure.

Consistent with the results of the phylogenetic relationships and comparison of estimated divergence times, all tRNA anticodons of the sequenced mitochondrial genomes of *C*. *chinensis* and *Curculio* sp. were identical to other Curculionini species (Tables 1 and 2). Of the 22 total tRNA genes, 14 are located on the N-strand and eight are located on the J-strand. Individual tRNAs of *C*. *chinensis* (MG728094) and *Curculio* sp. (MG728095) ranged from 63 bp (trnH) to 71 bp (trnK) in length. Secondary structure models of the tRNA genes from the two mitochondrial genomes were predicted using the Mitos WebServer (http://mitos.bioinf.uni-leipzig.de/). All tRNA genes from *C*. *chinensis* and *Curculio* sp. mitochondrial genomes fold into a canonical clover-leaf structure (Fig. 5).

The dihydrouridine (DHU) arm of all the tRNAs was a large loop, instead of the conserved stem-and-loop structure, which is consistent with typical metazoan mitochondrial genomes^40^. While the amino acid acceptor stem was conserved across 7 bp in all tRNA genes, the anticodon loops exhibited differences. trnH and trnR were conserved across 8 bp, while the rest of the 20 tRNAs were conserved across 7 bp (Fig. 5). The DHU arms in the tRNAs from *C*. *chinensis* and *Curculio* sp. were 0 to 4 bp long. The AC arms were 4 to 5 bp long, and the TΨC arms varied in length from 3 to 5 bp. The variable loops ranged from 4 to 8 bp. We also compared the variation in stem regions of tRNA genes among five other Curculionini species (Fig. 5). Among the 22 tRNA genes, trnI was the most conserved, and lacked nucleotide variation in stem regions between *C*. *chinensis* and *Curculio* sp. The rest of the tRNAs exhibited between 1–10 site mutations. The trnF had the highest number of site mutations on stem regions (10 sites), followed by trnV (7 sites) (Fig. 5). Among the 22 tRNA genes, there was no nucleotide variation in the stem regions between *Curculio davidi* (KY053741) and *Curculio davidi* (NC034931) (Fig. 5). *Curculio elephas* (KY0872691) had the most nucleotide variation in stem regions compared with *C*. *chinensis* (Fig. 5). Base pairs other than canonical A-Us and C-Gs were occasionally used in *C*. *chinensis* and *Curculio* sp. tRNAs, which is based on predicted tRNA secondary structures (Fig. 5). We found 22 and 15 mismatched base pairs in the tRNAs from *C*. *chinensis* and *Curculio* sp., respectively. Among the 22 mismatched base pairs in *C*. *chinensis*, three were U-U pairs, located in the amino acid acceptor stems and anticodon arm stems. The others were A–C pairs located in the amino acid acceptor stem. *Curculio* sp. had four U-U pairs t located in the TΨC stems (Fig. 5).

## Methods

### Sample collection and dna extraction

Camellia weevil samples were collected from Tengchong County in Yunnan Province, China. The field collected samples were initially placed in 100% ethyl alcohol and stored at −80 °C prior to DNA extraction. Total genomic DNA was extracted separately from the whole body of individual samples using a DNeasy tissue kit (Qiagen, Hilden, Germany). Voucher DNA was deposited in the entomological collections of the Research Institute of Subtropical Forestry, Chinese Academy of Forestry.

### Mitochondrial genome sequencing and assembly

The mitochondrial genome sequences were obtained by next-generation sequencing. Prior to library construction, the DNA was quantified by Qubit 3.0 (Invitrogen, Life technologies, Carlsbad, CA, USA)^5^. The library (Lib. Type: PE400; Lib. Insert Size: 400 bp) with two indexes was constructed using the Illumina TruSeq@ DNA PCR-Free HT Kit and sequenced by Shanghai Personal Biotechnology CO., Ltd (Shanghai, China) using Illumina Miseq with the strategy of 251 bp paired-ends by paired sequencing mode. Raw sequence reads were generated on the Illumina Miseq sequencing platform in FASTQ format, and read quality was evaluated using the FastQC software package (http://www.bioinformatics.babraham.ac.uk/projects/fastqc)^52^. Reads containing ambiguous nucleotides and reads with an average quality value lower than Q30 were excluded from further analyses. The high-quality second-generation sequencing data were assembled *de novo* to generate contig and scaffold sequences using the A5-miseq v.20150522^53^ and SPAdes v.3.9.0^54^ assembly pipelines. According to the sequencing depth extraction sequence of the splicing sequence, the high sequencing depth was blastn with the NT library in NCBI (BLAST v2.2.31+) and compared with the mitochondrial sequence of each splicing result. The mitochondrial splicing results were combined using the Mummer v.3.1 software to integrate splicing results. Linear analysis was used to determine the positions between contigs and fill gaps between contigs using the Pilon v.1.18 software package^55^. The results were then corrected to obtain the final mitochondrial genome sequences.

### Mitochondrial genome annotation

The complete set of linear contigs was uploaded to the MITOS web page server (http://mitos2.bioinf.uni-leipzig.de/) for functional annotation^56^. The optional setting for ‘Genetic Code’ was selected as 05-verterbrate, and the remaining settings were set according to the default parameters. The circular mitogenomes of both samples were visualized using the Organellar Genome Draw web server tool (http://ogdraw.mpimp-golm.mpg.de/)^57^. The sequin file generated from MITOS was edited and submitted to NCBI according to the ORF Finder results (NCBI GenBank accession number MG728094, MG728095).

### Comparative analysis of mitochondrial genomes

The mitochondrial genomes of five Curculionini species, including the two newly sequenced *Curculio* genomes, were compared. Gene arrangement, base composition, and PCG codon usage features were analyzed. We also analyzed base compositional differences based on the secondary structures of tRNA genes among the mitochondrial genomes of the five species. The AT- and GC-skew were calculated using the following formulas: AT-skew = (A% − T%)/(A% + T%) and GC-skew = (G% − C%)/(G% + C%)^38^. Intergenic spacers and overlapping regions between genes were manually counted. The rate of protein conformance among the 13 PCGs was analyzed using DNAMAN. K_a_ and K_s_ substitution values were calculated using the DNaSP V5.10^58^. Multiple protein structure prediction web servers (https://www.predictprotein.org/ and http://www.prabi.fr/)^59^ were used to predict the secondary structure of the ATP8 protein: The amino acid composition and coding sequence composition of the protein, combined with regional, screw, spiral transmembrane regions, and other irregular regions were analyzed. The protein loci of potential exposure areas and hidden areas were also predicted^60,61^.

### Phylogenetic analyses

Substitution saturation of different genes was tested in DAMBE5 using the GTR substitution model as a reference^62,63^. The best model of evolution for all genes and protein sequences was the GTR + I + G model, as determined by the jModelTest software package^64^. For the phylogenetic analysis, 55 published mitochondrial genomes were downloaded from NCBI as references and used along with the two *Curculio* sequenced mitochondrial genomes (Table S1).

Phylogenetic analyses incorporated both the Bayesian inference method (BI) using the program MRBAYES version 3.152^65^. Maximum Likelihood (ML) methods used the CIPRES server RAxML online (www.phylo.org). We used a Bayesian framework based on the PCG data to estimate the divergence times of clades using the BEAST v.1.6.1 software package. The substitution model (GTR + I + G) was also used for these analyses, as determined to best model the data by Jmodeltest^64^. The analysis was conducted with an expansion growth model and an uncorrelated lognormal relaxed clock, with a proposed insect molecular clock. Rates of nucleotide substitution were 10^−2^ subs/s/my/l for each mitochondrial protein-coding gene. The mean rate was 1.115 while the lower rate was 0.747 and the upper rate was 1.523^13^. Markov chains were analyzed three times for 500,000,000 generations, with sampling every 1,000 generations. The Tracer v.1.5 software package was used to verify the posterior distribution and the effective sample sizes (ESSs) from the MCMC output to ensure that the values were greater than 200. The chain analysis process was tested three times to ensure data stability. The Tree Annotator v1.7.5 component within the BEAST package^66^ was used to summarize a burn-in of 25% trees after the stationary chain likelihood values were established. The phylogenetic trees were viewed and edited using the FigTree software package.

## Supplementary information

Dataset 1
