# Supplementary material for: The complete mitochondrial genomes of two sibling species of camellia weevils (Coleoptera: Curculionidae) and patterns of Curculionini speciation
Source: Sci Rep. 2019 Mar 4;9:3412. doi: 10.1038/s41598-019-39895-8 (PMC6399312; doi:10.1038/s41598-019-39895-8)

**The complete** **mitochondrial genomes of two sibling species of camellia weevils (Coleoptera: Curculionidae) and patterns of** **Curculionini speciation**

**Shou-ke Zhang^1, 2^, Jin-ping Shu**^*^**^, 2^, Yang-dong Wang**^*^**^, 1, 2^, Ya-ning Liu^2^, Han Peng^2^, Wei Zhang^2^ & Hao-jie Wang^2^**

^1^State Key Laboratory of Tree Genetics and Breeding, Chinese Academy of Forestry, No.1, Dongxiaofu Xiangshan Road, Haidian District, Beijing 100091, P. R. China.

^2^Research Institute of Subtropical Forestry, Chinese Academy of Forestry, No.73, Daqiao Road, Fuyang District, Hangzhou, Zhejiang 311400, P. R. China.

^*^ Responding author:

Jin-ping Shu. Tel: +86 571 63329652, E-mail: shujinping_001@163.com

Yang-dong Wang. Tel: +86 57163100572, E-mail: Wyd11111@126.com

^*^Correspondence and requests for materials should be addressed to J.P.S. ( )

**Table S1. Mitochondrial genomes used in this study.**

| **Group** | **Superfamily** | **Family** | **Species** | **Accession Number** | **Nation** | **Reference** | |
| --- | --- | --- | --- | --- | --- | --- | --- |
| Ingroup | Curculionidae | Molytinae | *Hylobitelus xiaoi* | JX847496 | China | Zhou *et al*., 2013 | |
|  |  | Cryptorhynchinae | *Eucryptorrhynchus brandti* | KM593905 | China | Nan *et al*., 2014 | |
|  |  |  | *Eucryptorrhynchus chinensis* | KP410324 | China | Liu *et al*., 2015 | |
|  |  |  | *Eucryptorrhynchus brandti* | KP455482 | China | Liu *et al*., 2015 | |
|  |  |  | *Eucryptorrhynchus chinensis* | KP455510 | China | Nan *et al*., 2015 | |
|  |  |  | *Eucryptorrhynchus brandti* | NC025945 | USA | Nan *et al*., 2014 | |
|  |  |  | *Eucryptorrhynchus chinensis* | NC026719 | China | Liu *et al*., 2015 | |
|  |  | Curculioninae | *Curculio elephas* | KX087269 | UK | Hunter *et al*., 2017 | |
|  |  |  | *Curculio chinensis* | MG728094 | China | This paper | |
|  |  |  | *Curculio* sp. | MG728095 | China | This paper | |
|  |  |  | *Curculio davidi* | KY057374 | China | Guan *et al*., 2017 | |
|  |  |  | *Curculio davidi* | NC034293 | China | Guan *et al*., 2017 | |
|  |  | Cyclominae | *Aegorhinus superciliosus* | KF785807 | Chile | Cabrera-Brandt *et al*., 2015 | |
|  |  |  | *Aegorhinus superciliosus* | NC027577 | USA | Cabrera-Brandt *et al*., 2015 | |
|  |  | Dryophthorinae | *Rhynchophorus ferrugineus* | KT428893 | China | Bi *et al*., 2015 | |
|  |  |  | *Sitophilus zeamais* | KX373614 | USA | Ojo *et al*., 2016 | |
|  |  |  | *Sitophilus oryzae* | KX373615 | USA | Ojo *et al*., 2016 | |
|  |  |  | *Rhynchophorus ferrugineus* | NC028535 | China | Bi *et al*., 2015 | |
|  |  |  | *Sitophilus zeamais* | NC030764 | UK | Ojo *et al*., 2016 | |
|  |  | Entiminae | *Naupactus xanthographus* | GU176345 | USA | Song *et al*., 2010 | |
|  |  |  | *Sympiezomias velatus* | MF383367 | China | Tang *et al*., 2017 | |
|  |  |  | *Sitona callosus* | MF594624 | China | Zhang *et al*., 2017 | |
|  |  | Scolytinae | *Xylosandrus morigerus* | KX035191 | UK | Miller *et al*., 2017 | |
|  |  |  | *Trypophloeus asperatus* | KX035204 | UK | Miller *et al*., 2017 | |
|  |  |  | *Trypodendron domesticum* | KX035205 | UK | Miller *et al*., 2017 | |
|  |  |  | *Dryocoetes autographus* | KX035207 | UK | Miller *et al*., 2017 | |
|  |  |  | *Hylastes brunneus* | KX035208 | UK | Miller *et al*., 2017 | |
|  |  |  | *Pityophthorus pubescens* | KX035209 | UK | Miller *et al*., 2017 | |
|  |  |  | *Pityogenes bidentatus* | KX035211 | UK | Miller *et al*., 2017 | |
|  |  |  | *Hylastes attenuatus* | KX035212 | UK | Miller *et al*., 2017 | |
|  |  |  | *Orthotomicus laricis* | KX035213 | UK | Miller *et al*., 2017 | |
|  |  |  | *Trypodendron signatum* | KX035214 | UK | Miller *et al*., 2017 | |
|  |  |  | *Anisandrus dispar* | KX035217 | UK | Miller *et al*., 2017 | |
|  |  |  | *Gnathotrichus materiarius* | KX035218 | UK | Miller *et al*., 2017 | |
|  |  |  | *Cyclorhipidion bodoanus* | KX035219 | UK | Miller *et al*., 2017 | |
|  |  |  | *Hypothenemus sp.* | KX035224 | UK | Miller *et al*., 2017 | |
|  |  |  | *Pityogenes trepanatus* | KX035225 | UK | Miller *et al*., 2017 | |
|  |  |  | *Tomicus piniperda* | KX035226 | UK | Miller *et al*., 2017 | |
|  |  |  | *Hylastes brunneus* | NC036262 | UK | Miller *et al*., 2017 | |
|  |  |  | *Xylosandrus germanus* | NC036280 | UK | Miller *et al*., 2017 | |
|  |  |  | *Ips sexdentatus* | NC036281 | UK | Miller *et al*., 2017 | |
|  |  |  | *Dryocoetes villosus* | NC036282 | UK | Miller *et al*., 2017 | |
|  |  |  | *Xylosandrus morigerus* | NC036283 | UK | Miller *et al*., 2017 | |
|  |  |  | *Xylosandrus crassiusculus* | NC036284 | UK | Miller *et al*., 2017 | |
|  |  |  | *Trypophloeus asperatus* | NC036285 | UK | Miller *et al*., 2017 | |
|  |  |  | *Dryocoetes autographus* | NC036287 | UK | Miller *et al*., 2017 | |
|  |  |  | *Pityophthorus pubescens* | NC036288 | UK | Miller *et al*., 2017 | |
|  |  |  | *Pityogenes bidentatus* | NC036289 | UK | Miller *et al*., 2017 | |
|  |  |  | *Hylastes attenuatus* | NC036290 | UK | Miller *et al*., 2017 | |
|  |  |  | *Orthotomicus laricis* | NC036291 | UK | Miller *et al*., 2017 | |
|  |  |  | *Trypodendron signatum* | NC036292 | UK | Miller *et al*., 2017 | |
|  |  |  | *Anisandrus dispar* | NC036293 | UK | Miller *et al*., 2017 | |
|  |  |  | *Gnathotrichus materiarius* | NC036294 | UK | Miller *et al*., 2017 | |
|  |  |  | *Cyclorhipidion bodoanus* | NC036295 | UK | Miller *et al*., 2017 | |
| Outgroup | Leamophloeidae |  | *Cryptolestes pusillus* | KT070713 | China | | Li *et al*., 2015 |

**Figure S1.** Map and rearrangement of the mitochondrial genomes in *Curculio chinensis* (MG728094) and *Curculio* sp. (MG728095).


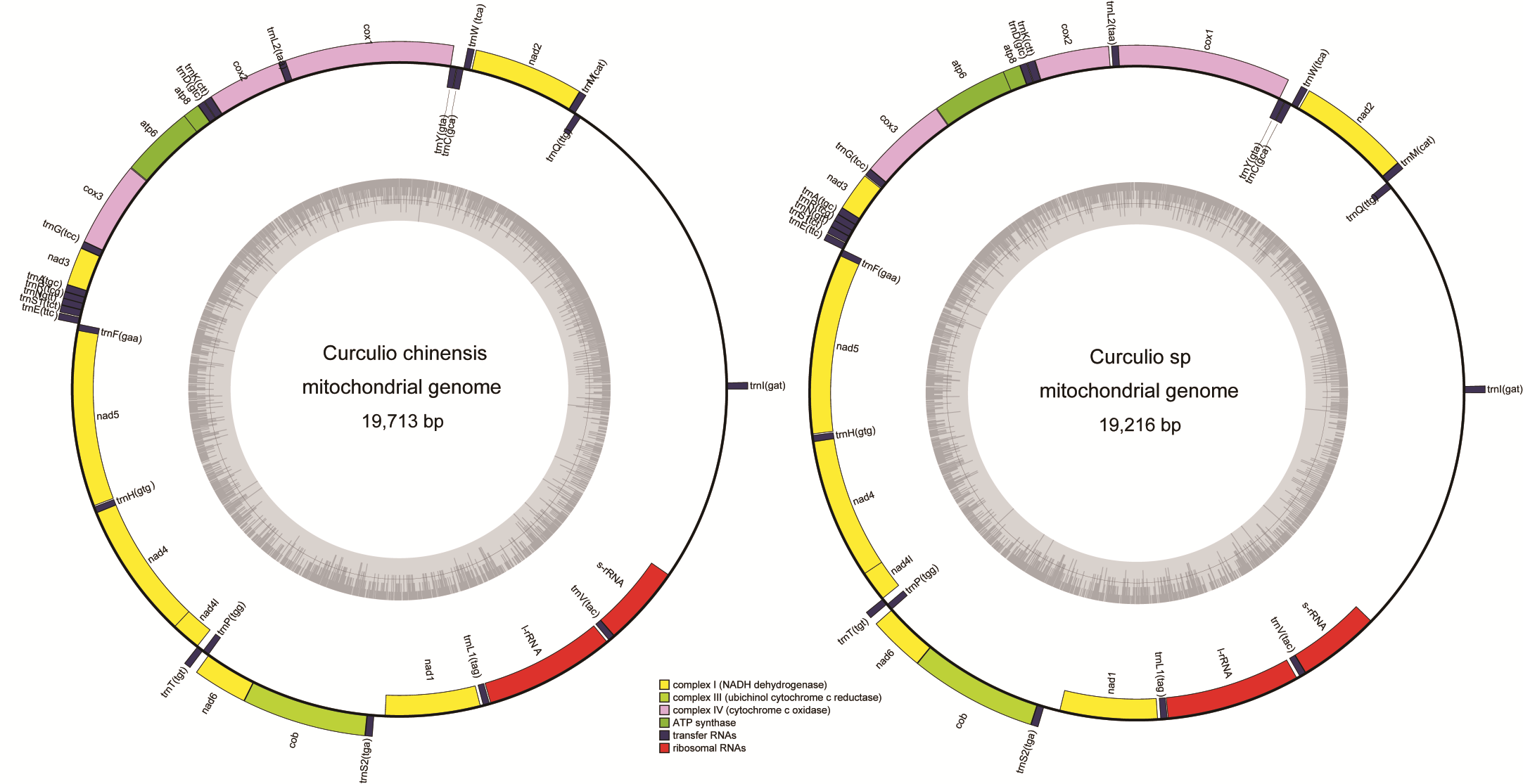

Supplement: Supplementary file 1 — Dataset 1 [file 41598_2019_39895_MOESM1_ESM.doc]
